# Promoting a Patient Safety Culture in Anesthesia Practice

**DOI:** 10.5812/aapm-154453

**Published:** 2024-11-25

**Authors:** Maryam Vosoughian, Sara Salarian, Mastaneh Dahi Taleghani

**Affiliations:** 1 Department of Anesthesiology & Critical Care, Anesthesiology Research Center, Taleghani Hospital, Shahid Beheshti University of Medical Sciences, Tehran, Iran; 2 Department of Anesthesiology & Critical Care, Anesthesiology Research Center, Emam Hosein Hospital, Shahid Beheshti University of Medical Sciences, Tehran, Iran

**Keywords:** Anesthesia, Culture, Patient Safety

*Dear Editor,*

With the rapid advancement of technology and medical sciences, patient safety has emerged as a global priority, requiring meticulous attention and strategic interventions (1). Adverse events impact 10% of hospital admissions in developing countries, resulting in increased costs, injuries, disabilities, and deaths (2). The specialty of anesthesiology has been a leader in medicine over the past half-century, pursuing patient safety research and implementing standards of care and systematic improvements in care processes. Building a robust safety culture is essential for improving patient safety. This involves fostering a rewarding approach to reporting errors, promoting teamwork, documenting mistakes, and analyzing errors to learn from them (3). However, in Iran, there appear to be areas for improvement in establishing this culture, particularly within the field of anesthesia (4, 5).

To address this, the Department of Anesthesiology at Shahid Beheshti University has initiated a program aimed at encouraging residents to voluntarily report errors in anesthesia management. By implementing a system where reported errors are documented on designated websites, we aim to incentivize participation by increasing the scores of serial and monthly examinations for those who contribute.

While this approach may create an inflated perception of error rates, we believe the advantages of fostering open discussions about safety far outweigh the drawbacks. This initiative strives to establish a transparent environment where residents feel empowered to report mistakes without fear of retribution, ultimately strengthening the culture of patient safety.

We hope that sharing our experiences and strategies will inspire further dialogue on improving patient safety practices in anesthesia worldwide.
